# ACCELERATED BIOLOGICAL AGE IS ASSOCIATED WITH DELIRIUM AND PLASMA NEUROFILAMENT LIGHT IN GERIATRIC HIP FRACTURE

**DOI:** 10.1093/geroni/igad104.0387

**Published:** 2023-12-21

**Authors:** Sara LaHue, Joy Youn, Matias Fuentealba, Vanja Douglas, David Furman, Julio Rojas, Lawren VandeVrede, John Newman

**Affiliations:** University of California, San Francisco, San Francisco, California, United States; UCSF, San Francisco, California, United States; Buck Institute, Novato, California, United States; UCSF, San Francisco, California, United States; Buck Institute, Novato, California, United States; UCSF, San Francisco, California, United States; UCSF, San Francisco, California, United States; Buck Institute, Novato, California, United States

## Abstract

**Background:**

Biological age may be distinct from chronological age. Epigenetic clocks (e.g., PhenoAge) estimate biological age by quantifying changes in DNA methylation (DNAm). Biological age is “accelerated” (AgeAccel) when epigenetic>chronological age. AgeAccel predicts age-related diseases but its association with delirium or neuronal injury markers (e.g., neurofilament light “NfL”) is unknown.

**Methods:**

Adults age 65+ hospitalized for acute hip fracture underwent daily delirium screening with the Confusion Assessment Method Long Form. DNAm status of 850,000 CpG sites was measured in triplicate from pre-op peripheral blood mononuclear cells using Illumina MethylationEPIC arrays. AgeAccel represented the residual of the linear regression model of PhenoAge regressed on chronological age. Plasma NfL was measured in duplicate using Simoa immunoassays. Group differences calculated by T-test.

**Results:**

Of 12 subjects (enrollment ongoing): mean age 79±8, 75% women, 42% with dementia, 33% were delirious on pre-op blood collection day. Mean AgeAccel was 4.4 years (p=0.02) in delirious vs non-delirious subjects (Fig1A). Those with positive (vs negative) AgeAccel had higher mean NfL (Fig1B, p=0.002). Delirious (vs non-delirious) subjects had higher mean NfL (p=0.004). We found no difference in either AgeAccel or NfL by dementia status.

**Conclusions:**

In this geriatric hip fracture pilot, accelerated biological age was associated with higher delirium prevalence and NfL. Delirium was also associated with higher NfL. This pilot demonstrates feasibility and utility of measuring biological aging in delirium that warrants study in a larger cohort.

